# Upfront Impella CP as Bridge-to-Transcatheter Aortic Valve Replacement in Severe Aortic Stenosis and Cardiogenic Shock: Case Report

**DOI:** 10.1016/j.jscai.2025.103924

**Published:** 2025-09-16

**Authors:** Dorian Garin, Benjamin Assouline, Sophie Degrauwe, Juan F. Iglesias

**Affiliations:** a Department of Cardiology, Geneva University Hospitals, Geneva, Switzerland; b Department of Intensive Care Unit, Geneva University Hospitals, Geneva, Switzerland

**Keywords:** aortic stenosis, cardiogenic shock, case report, circulatory support device, transcatheter aortic valve replacement

## Abstract

A 75-year-old woman presented with severe aortic stenosis and CS (ejection fraction, 10% to 15%; lactate, 4.2 mmol/L; requiring high-dose vasopressors). During index catheterization, upfront Impella CP insertion across the stenotic valve immediately improved hemodynamics (peak-to-peak gradient reduced from 125 to 6 mm Hg). Following stabilization, staged TAVR was performed within 15 hours through the same femoral access. The patient was discharged on day 12 with complete left ventricular recovery (ejection fraction, 65% to 70%) at 6 months. This represents the first reported case of upfront Impella CP insertion during index procedure as bridge-to-TAVR without balloon aortic valvuloplasty, demonstrating feasibility and excellent outcomes in AS-related CS.

## Introduction

Severe aortic stenosis (AS) complicated by cardiogenic shock (CS) represents a clinical challenge, with mortality rates exceeding 50% with medical management alone. Emergency surgical aortic valve replacement carries prohibitive risk in hemodynamically unstable patients.^1^ Balloon aortic valvuloplasty (BAV) as bridge-to–aortic valve replacement shows poor outcomes with 30-day mortality rates of 25% to 40%.^2^ Transcatheter aortic valve replacement (TAVR) has emerged as an alternative but remains associated with significant morbidity and mortality in patients with CS, with 30-day mortality rates ranging from 20% to 42%.^3^

The Impella CP microaxial flow pump (Abiomed) has demonstrated improved outcomes in acute myocardial infarction–related CS,^4^ but evidence in valvular heart disease remains limited. While TAVR under mechanical circulatory support (MCS) has been reported in CS induced by AS.^1^^,^^3^ MCS devices were typically implanted in a staged manner after initial medical stabilization or BAV.^5^ The upfront use of Impella CP as first-line bridge therapy during index catheterization to facilitate early TAVR without BAV has not been described.

## Case presentation

**Figure 1:**
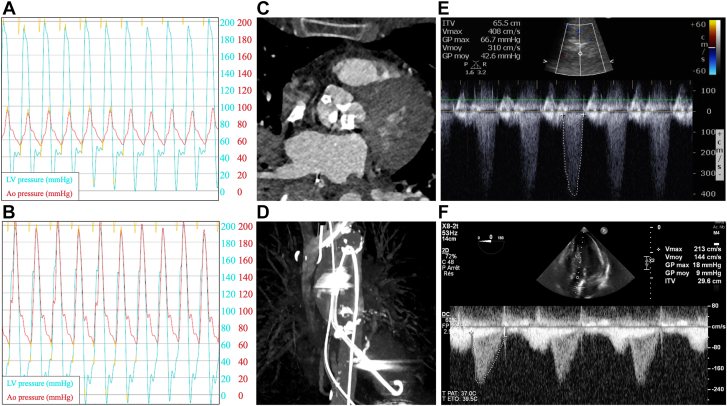
**Hemodynamic and imaging data during Impella-supported bridge-to-TAVR.** (**A**) Pre-Impella invasive pressure tracings showing severe peak-to-peak gradient of 125 mm Hg. (**B**) Post-Impella pressure tracings demonstrating immediate gradient reduction to 6 mm Hg. (**C**) Axial and (**D**) maximum intensity projection cardiac computed tomography with Impella CP correctly positioned across aortic valve into left ventricle. (**E**) Pre-TAVR transthoracic echocardiography showing a low-flow high-gradient aortic stenosis. (**F**) Post-TAVR transthoracic echocardiography showing complete recovery, with ejection fraction of 65%-70% and normal prosthetic valve function. TAVR, transcatheter aortic valve replacement.

A 75-year-old woman with no significant medical history presented at 1 am with acute chest pain and progressive dyspnea over 6 hours. She rapidly deteriorated with acute pulmonary edema requiring emergent intubation and high-dose vasopressor support for profound CS (hemodynamic details in Table 1). Bedside echocardiography revealed severe left ventricular (LV) dysfunction (ejection fraction, 10% to 15%; Figure 1E) with global hypokinesis and severe AS (valve area, 0.57 cm^2^; mean gradient, 42 mm Hg). Emergency coronary angiography excluded coronary disease. Left heart catheterization revealed markedly elevated LV end-diastolic pressure (50 mm Hg) and peak-to-peak aortic gradient (125 mm Hg) (Figure 1A).

**Table 1:** Hemodynamic and metabolic parameters throughout hospitalization and follow-up.

Parameter	Baseline	Post-Impella	Pre-TAVR (Impella in situ)	Post-TAVR	6-mo follow-up
Hemodynamics					
Systolic BP, mm Hg	70	95	122	150	133
Heart rate, bpm	125	103	93	72	83
PCWP, mm Hg	50	36	25	18	–
Peak-to-peak gradient, mm Hg	125	6	–	10	–
Lactate, mmol/L	4.2	3.0	–	1.2	–
Norepinephrine, μg/kg/min	0.08	0.06	0.02	0	0
Dobutamine, μg/kg/min	6	4	2	0	0
Echocardiographic parameters					
LVEF, %	10-15	25	Moderately reduced^a^	35	65-70
LV diameter, cm	5.4	5.1	–	3.8	4.2
LV geometry^b^	Eccentric hypertrophy	Eccentric hypertrophy	–	Concentric hypertrophy	Concentric remodeling
Aortic valve					
AVA, cm^2^^c^	0.57^c^	–	–	2.1	2.1
AVA indexed, cm^2^/m^2^^c^	0.32	–	–	1.2	1.2
Mean gradient, mm Hg	42	–	–	10	9
Peak gradient, mm Hg	69	–	–	13	12
Maximum velocity, cm/s	408	–	–	181	175
DVI	0.15	–	–	0.78	0.80
AR grade	–	–	Mild^a^	Trace	Minimal
Other parameters					
RV function	Normal	Normal	Normal^a^	Normal	Normal
MR grade	–	–	–	–	Nonsignificant
TR grade	–	–	–	–	Nonsignificant

AR, aortic regurgitation; AVA, aortic valve area; BP, blood pressure; bpm, beats per minute; DVI, Doppler velocity index; LV, left ventricular; LVEF, left ventricular ejection fraction; MR, mitral regurgitation; PCWP, pulmonary capillary wedge pressure; RV, right ventricular; TAVR, transcatheter aortic valve replacement; TR, tricuspid regurgitation.

a Measured by transesophageal echocardiography. Other measurements were performed by transthoracic echocardiography.

b According to American Society of Echocardiography criteria.

c According to continuity equation.

During catheterization, hemodynamic instability persisted despite escalation of medical therapy. After discussion with the intensive care team, a decision was made for upfront Impella CP insertion based on its ability to provide immediate LV unloading while serving as bridge to definitive therapy. The Impella CP was inserted via 14F catheter right femoral access. Despite severe AS, the device successfully crossed the stenotic valve without requiring BAV. The device was set to performance level P8, allowing a flow rate of 3.8 L/min. Correct positioning was confirmed via radioscopy, pressure waveforms, and transthoracic echocardiography. Immediate hemodynamic improvement occurred, with the peak-to-peak gradient reduced from 125 to 6 mm Hg (Figure 1B), allowing for the progressive weaning of vasopressors (Table 1). The next day, cardiac computed tomography was performed with Impella in situ (Figure 1C, D), revealing adequate vascular and valve anatomy for TAVR. An emergent heart team meeting recommended TAVR after surgical consultation deemed operative risk prohibitive (EuroSCORE II, 24.9%).

Fifteen hours after admission, the Impella CP was removed; predilation was performed with a 22.0-mm Z-MED II balloon (NuMED) allowing for a 29.0-mm Evolut PRO+ (Medtronic) to be implanted through the same femoral access. Postdilation was performed with the same balloon. Postdeployment hemodynamics showed residual peak-to-peak gradient of 10 mm Hg with no significant paravalvular leak. The patient was extubated within 24 hours and discharged on day 12.

**Figure 2:**
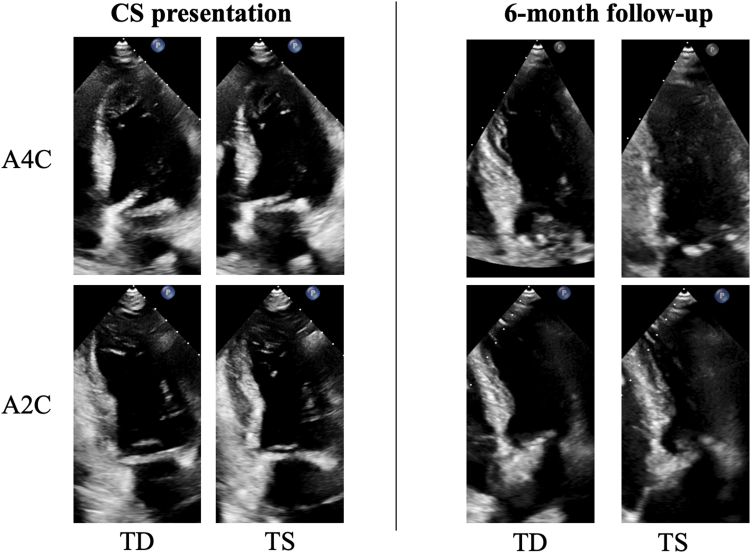
**Echocardiographic demonstration of reversible left ventricular dysfunction.** Initial transthoracic echocardiography in A4C and A2C views showing severe left ventricular dysfunction with ejection fraction of 10%-15%. Follow-up transthoracic echocardiography in A4C view showing complete recovery with ejection fraction of 65%-70% and normal prosthetic valve function. A2C, apical 2-chamber view; A4C, apical 4-chamber view; CS, cardiogenic shock; TD, telediastolic; TS, telesystolic.

At 6-month follow-up, the patient remained at New York Heart Association class I with complete LV recovery (ejection fraction, 65%-70%) (Figure 2) and normal prosthetic valve function (Figure 1F).

## Discussion

This case represents, to our knowledge, the first documented upfront Impella CP insertion as primary bridge strategy enabling staged TAVR without BAV in severe AS with CS. While previous reports describe MCS during TAVR or after initial stabilization with BAV,^2^^,^^5^ our approach demonstrates feasibility of immediate Impella support during index catheterization.

The absence of coronary disease, lack of regional wall motion abnormalities, and rapid hemodynamic improvement with afterload reduction confirmed severe AS with myocardial stunning from afterload mismatch rather than primary myocardial CS. During index catheterization, we selected Impella CP over alternative MCS options based on its unique advantages: unlike intra-aortic balloon pump (contraindicated in AS), venoarterial extracorporeal membrane oxygenation (increases afterload^1^), or TandemHeart (LivaNova) (requires transseptal puncture), Impella provides immediate LV unloading while maintaining cardiac output.^6^ Direct emergent TAVR was not feasible given nocturnal presentation without plateau technique availability.

Although severe AS has traditionally contraindicated Impella use, emerging evidence supports its feasibility.^7^^,^^8^ While valve injury has been reported,^9^ this concern becomes less relevant when valve replacement is already mandated. Our strategic approach deliberately awaited hemodynamic stabilization under Impella support before TAVR, allowing end-organ recovery and optimization of loading conditions. Technical precautions included maintaining guide wire position, rapid exchange technique, and standby surgical backup. The absence of hemodynamic compromise following Impella removal validates this bridge-to-TAVR strategy.

This staged approach offers advantages over alternatives: BAV provides only temporary relief, while emergent TAVR in profound CS carries prohibitive risk.^1^ The 15-hour stabilization period enabled proper preprocedural planning and elective TAVR. Using the same femoral access minimized vascular complications. Complete LV recovery at 6 months suggests early MCS implementation prevented irreversible myocardial damage, extending the concept of preprocedural support from acute myocardial infarction–related CS^4^^,^^10^ to valvular CS.

The optimal timing between Impella insertion and TAVR requires further study, balancing stabilization benefits against device-related complications. We recommend awaiting hemodynamic improvement once Impella is in place, as there is no urgency for TAVR once adequate circulatory support is established.

### Limitations

This single case report requires validation in larger series. Patient selection remains crucial, particularly regarding valve anatomy allowing safe device crossing, absence of significant coronary disease, adequate vascular access, and team experience with both MCS and TAVR. The Impella CP could be inserted without BAV despite the severely stenotic aortic valve; this may not be replicable in all cases. Additionally, using the same vascular access raises potential infection concerns.

## Conclusion

Upfront Impella CP during index catheterization as bridge-to-TAVR represents a feasible strategy for severe AS with CS, potentially improving outcomes in this high-risk population. This approach may expand treatment options for prohibitive risk patients traditionally denied intervention.

## Declaration of competing interest

The authors declared no potential conflicts of interest with respect to the research, authorship, and/or publication of this article.

## Funding sources

This work was not supported by funding agencies in the public, commercial, or not-for-profit sectors.

## Ethics statement and patient consent

Written informed consent was obtained from the patient for publication of this case report and accompanying images. The study was conducted in accordance with the Declaration of Helsinki and institutional review board approval was waived.
